# High dose of dexamethasone protects against EAE-induced motor deficits but impairs learning/memory in C57BL/6 mice

**DOI:** 10.1038/s41598-019-43217-3

**Published:** 2019-04-30

**Authors:** Nilton dos Santos, Leonardo S. Novaes, Guilherme Dragunas, Jennifer R. Rodrigues, Wesley Brandão, Rosana Camarini, Jean Pierre Schatzmann Peron, Carolina Demarchi Munhoz

**Affiliations:** 1 Department of Pharmacology, Institute of Biomedical Science, University of São Paulo, São Paulo, 05508-000 Brazil; 2 Department of Immunology, Institute of Biomedical Science, University of São Paulo, São Paulo, 05508-000 Brazil

**Keywords:** Multiple sclerosis, Molecular neuroscience

## Abstract

Multiple sclerosis (MS) is an autoimmune and neuroinflammatory disease characterized by demyelination of the Central Nervous System. Immune cells activation and release of pro-inflammatory cytokines play a crucial role in the disease modulation, decisively contributing to the neurodegeneration observed in MS and the experimental autoimmune encephalomyelitis (EAE), the widely used MS animal model. Synthetic glucocorticoids, commonly used to treat the MS attacks, have controversial effects on neuroinflammation and cognition. We sought to verify the influence of dexamethasone (DEX) on the EAE progression and on EAE-induced cognitive deficits. In myelin oligodendrocyte glycoprotein peptide (MOG35-55)-induced EAE female mice, treated once with DEX (50 mg/kg) or not, on the day of immunization, DEX decreased EAE-induced motor clinical scores, infiltrating cells in the spinal cord and delayed serum corticosterone peak. At the asymptomatic phase (8-day post-immunization), DEX did not protected from the EAE-induced memory consolidation deficits, which were accompanied by increased glucocorticoid receptor (GR) activity and decreased EGR-1 expression in the hippocampus. Blunting hippocampal GR genomic activation with DnGR vectors prevented DEX effects on EAE-induced memory impairment. These data suggest that, although DEX improves clinical signs, it decreases cognitive and memory capacity by diminishing neuronal activity and potentiating some aspects of neuroinflammation in EAE.

## Introduction

Human and animal studies have revealed a clear relationship between glucocorticoids (GCs) and some brain-related disorders, such as anxiety, depression, dementia, and schizophrenia^1,2^. Several brain regions, such as the medial prefrontal cortex (mPFC), hippocampus, and amygdala orchestrate cognitive functions in health and disease. In this context, morphological and activity modifications induced by endogenous or synthetic GC administration in these brain areas can change the hypothalamic-pituitary-adrenal (HPA) axis activity, causing anxiety, memory impairment and other brain disorders^3,4^.

High doses of synthetic GC, such as dexamethasone (DEX), prednisolone, and methylprednisolone, are commonly used to suppress immune activation and to treat the onset of the acute inflammatory symptomatic phase in relapse-remitting multiple sclerosis (MS-RRMS) patients, which becomes less effective across the MS clinical progression. GC can, indeed, alleviate the evolution of EAE in a dose-dependent manner^5^. However, GC (e.g., dexamethasone), whose metabolism in the organism ranges from 34 to 56 hours, can lead to worsening effects in the memory and locomotor activity^6^. In addition, several studies using different approaches showed the pro-inflammatory GC effects in the central nervous system (CNS), with increased NFKB activation, pro-inflammatory cytokines (IL1β, TNF-α) levels and reactive oxygen species (ROS) production, and inhibited neurogenesis in hippocampus and frontal cortex, both *in vivo* and *in vitro*^7–9^.

The EAE, an experimental model of MS, exhibits very similar histopathological and biomolecular markers^10,11^. Innate and acquired immune responses are implicated in both MS and EAE pathogenesis, including major histocompatibility complex (MHC) presentation of myelin-derived epitopes and cytokines release by dendritic cells and macrophages, activation of T lymphocytes (CD4^+^ and CD8^+^ T cells) into the perivascular region of the brain and spinal cord white matter, increased glial activation, and neurodegeneration^12,13^. Altogether, this implies in severe motor, sensory, autonomic, and neurocognitive clinical symptoms^13,14^.

Along with physical impairments, MS is also associated with behavioral, neuropsychiatric, and cognitive deterioration^15^. Indeed, 50% of MS patients are depressed^16^ and show cognitive deficits^17^; anxiety affects more than 35%, and chronic fatigue affects 50–60% of MS patients^18^. Recent experimental studies have shown that Biozzi ABH mice immunized with myelin oligodendrocyte glycoprotein (MOG_35-55_), which exhibits a remitting-relapsing EAE, presented hippocampal LTP (long-term potentiation) dysfunction, affecting aversive and non-aversive memory consolidation^19^. These effects on LTP and spatial memory impairment appeared to be dependent on IL-1β release and the oxidative stress induced by NADPH oxidase overexpression. In these settings, methylprednisolone treatment did not prevent the excitatory postsynaptic currents (EPSC) deficits in the hippocampus of immunized animals^19^. Also, EAE animals in the pre-symptomatic phases showed memory deficits in water maze tasks, which were dependent of TNF-α and IL1β mRNA overexpression in the hypothalamus but not in the amygdala or hippocampus of EAE mice^20^. These cytokines have a significant role in sterile inflammation and autoimmune diseases, contributing to the inflammatory cascade within CNS after EAE onset^21^.

In this sense, the MS and EAE-induced dysregulation of the HPA axis and neuroinflammation can be related to mood and cognitive symptoms^14,22^. Also, the treatment with synthetic GC could produce GR resistance through different mechanisms, such as (1) alternative splicing generating GRβ isoform in humans, (2) transactivation of others transcription factors, i.e., AP-1, and (3) modulation by non-classic kinases pathways, i.e., MAP kinases^23^. Furthermore, the augment of GC release by different types of stress can either protect^24^ or potentialize^25^ EAE progression.

This study aimed to elucidate the role of GCs signaling in the hippocampus after EAE- and DEX-induced cognitive impairment, focusing on the canonical GR signaling in CNS via recruitment of the CREB-EGR-1 pathway involved in neuronal activity and plasticity. Our results showed that although the treatment with DEX on the day of EAE induction has protected against motor signals progression, it induced HPA axis malfunction and diminished neuronal activity in the dorsal hippocampus, which was associated with memory loss related to EAE. These DEX effects were accompanied by a decreased CREB activity and EGR-1 expression during the disease.

## Results

### Dexamethasone protects against EAE clinical signs and delays the peak of serum corticosterone

**Figure 1:**
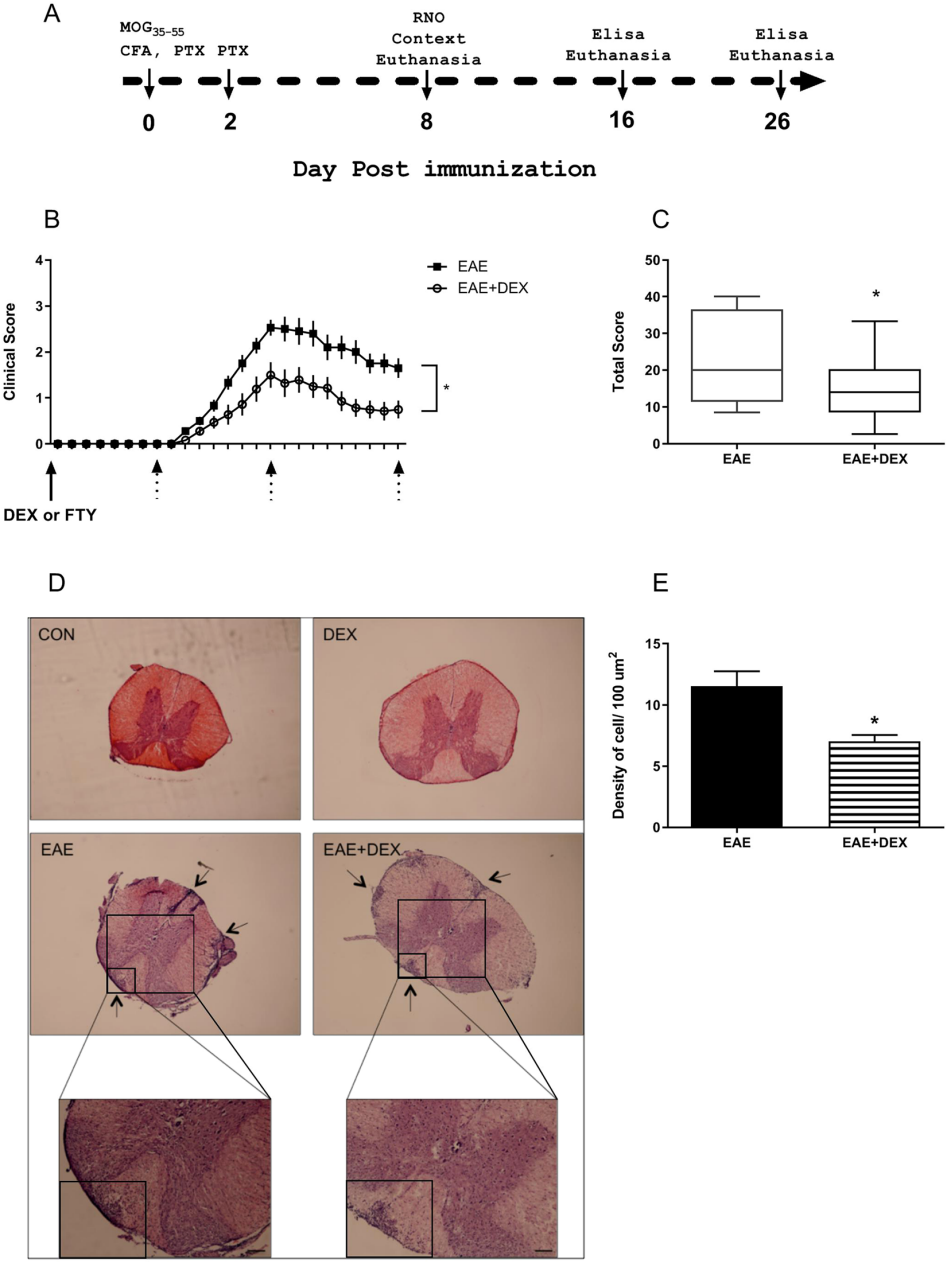
Evaluation of the clinical score over time of C57BL/6 female mice immunized with MOG_35-55_ emulsified with CFA, treated with Dexamethasone (DEX, 50 mg/kg) or Fingolimod (FTY-720, 10 mg/kg) at the day of immunization. (**A**) The schematic timeline for the experimental design. (**B**) Treatment with DEX on the day of immunization protected against EAE clinical score evolution; dotted arrows indicate the day of the euthanasia. (**C**) The sum of the total score from each animal at the 26 dpi was used to determine disease severity. (**D**) Hematoxylin-Eosin staining of mice spinal cord, arrows showed infiltrating clusters of peripheral cells in EAE and EAE + DEX. (**E**) Density of infiltrating cells in the white matter of EAE and EAE + DEX spinal cords/100 μm^2^. The results are showed as mean ± SEM of the experimental groups (N = 18). *p < 0.05 vs EAE [Two-way ANOVA followed by Bonferroni post-test (**B**), bi-caudal T-test (**C**) and Mann-Whitney non-parametric test (**E**)].

Schematic timeline for the experimental design of EAE is exposed in Fig. 1A. We observed the appearance of the clinical signs in the mice immunized with MOG_35-55_ in CFA (*Complete Freund Adjuvant)* at the 10-day post-immunization (dpi; Fig. 1B). The clinical scores peaked at the 16 dpi and partially remitted at the 26 dpi when the clinical scores from EAE mice were different from those in the EAE + DEX group (*F* = (*24,742)* = *5,113; interaction, p* = *0,0001*) (Fig. 1B).

DEX (50 mg/kg) treatment alone had no effect on the appearance of clinical signs. Despite both EAE and EAE + DEX groups began to present the motor deficits at the same day (10th dpi), the EAE + DEX group exhibited lower scores than EAE from the 13 dpi until the 26 dpi, showing that DEX attenuated the EAE motor signs development (*F* = (*17,170)* = *2,508; interaction, p* = *0,0014*) (Fig. 1B). Moreover, the sum of the scores in EAE + DEX animals was also lower than EAE during the disease (*p* = *0,0481*) (Fig. 1C). In addition to the attenuating effect on the clinical scores in EAE mice, this drug diminished the inflammatory cells infiltration in the spinal cord of diseased animals at the remission phase (*p* = *0,0397*) (26 dpi, Fig. 1D,E).

**Figure 2:**
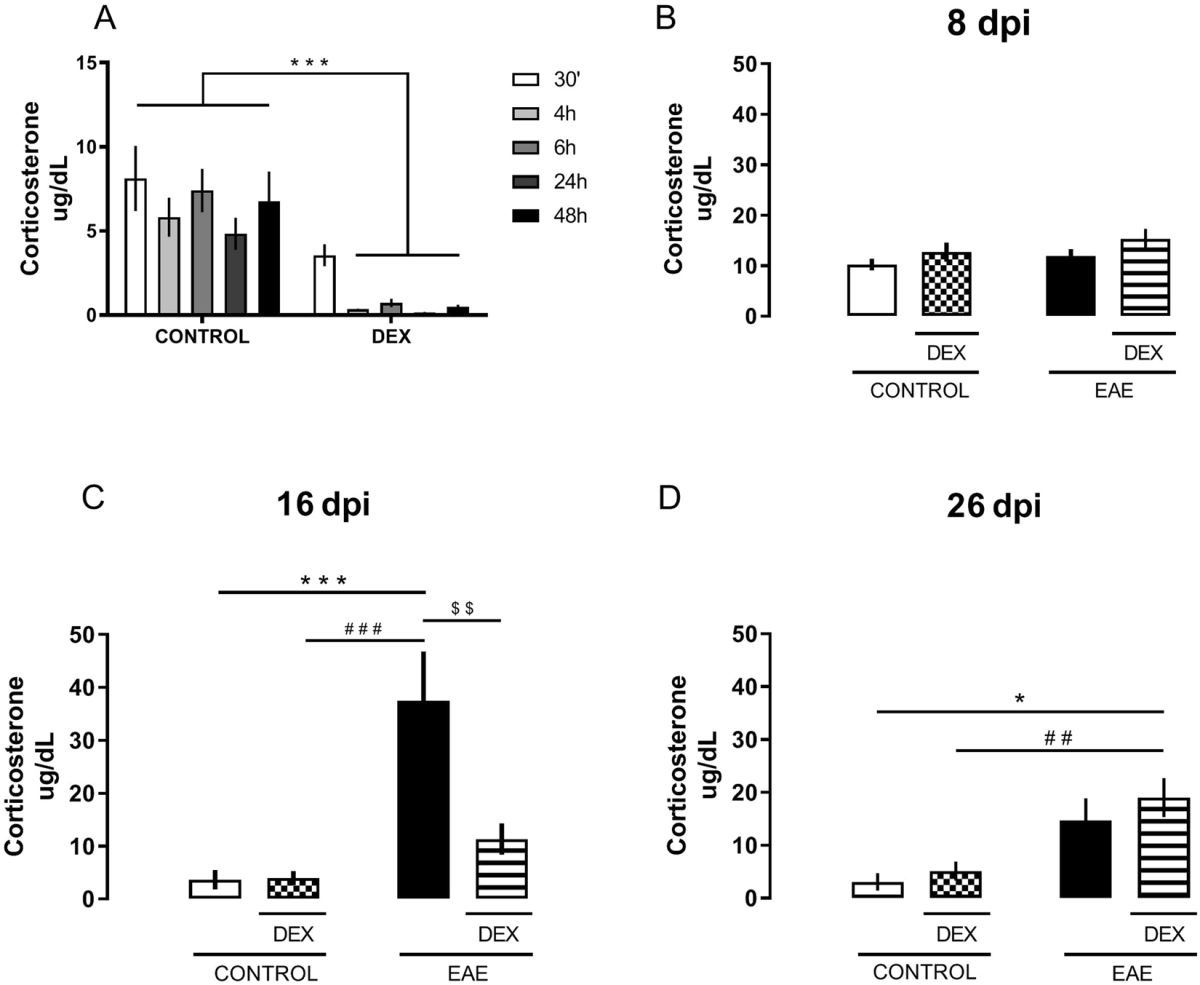
Dexamethasone treatment delays the peak of serum corticosterone levels in EAE animals. (**A**) Corticosterone time curve after DEX treatment: 30 min, 4 h, 6 h, 24 h, and 48 h. (**B**) There were no differences in serum corticosterone levels among all groups at the 8 dpi. (**C**) EAE increased serum corticosterone levels at the peak of disease (16 dpi), an effect prevented by DEX treatment. (**D**) EAE + DEX animals showed high levels of serum corticosterone when compared to the control group at the 26 dpi. The results are shown as mean ± SEM of the experimental groups. *p < 0.05 vs control, ^#^p < 0.05 vs DEX, and ^$^p < 0.05 vs EAE. Two-way ANOVA test followed by Bonferroni post-test, n = 4 to 6 for each group.

Several studies have shown HPA axis dysregulation in EAE and multiple sclerosis. Here, we observed that DEX indeed blunted corticosterone release up to 48 hours after its administration (*F* (*1,43*) = *75,29*; DEX effect, *p* < *0,0001*) (Fig. 2A), showing its suppressive effect on the HPA axis. No differences were found on the CORT serum levels at the 8 dpi (Fig. 2B) when all behavioral and biochemical analysis were made. As suggested in several studies, in EAE mice, we observed that serum corticosterone levels followed the magnitude of clinical scores, peaking at the 16 dpi (interaction, *F(1,14)* = *8,662; P* = *0,0107*) (Fig. 2C), reducing pursuant to the decreased severity of the motor deficits scores and returning to control levels at the 26 dpi (Fig. 2D). However, in DEX-treated EAE-induced mice, serum CORT peak was prevented at the 16 dpi (Fig. 2C), shifting to the 26 dpi [Dex effect, (*F(1,15)* = *21,57; P* = *0,0003*)] (Fig. 2D). These results suggest that DEX treatment changes the HPA axis activity throughout the disease.

### DEX did not protect against memory acquisition/consolidation deficits in EAE

In addition to the motor signs, both EAE-induced animals and MS patients show mood and memory disorders. Here, we tested whether DEX treatment would impact the working memory acquisition/consolidation in EAE-induced animals at the asymptomatic phase (7 and 8 dpi, before the onset of the clinical signs).

**Figure 3:**
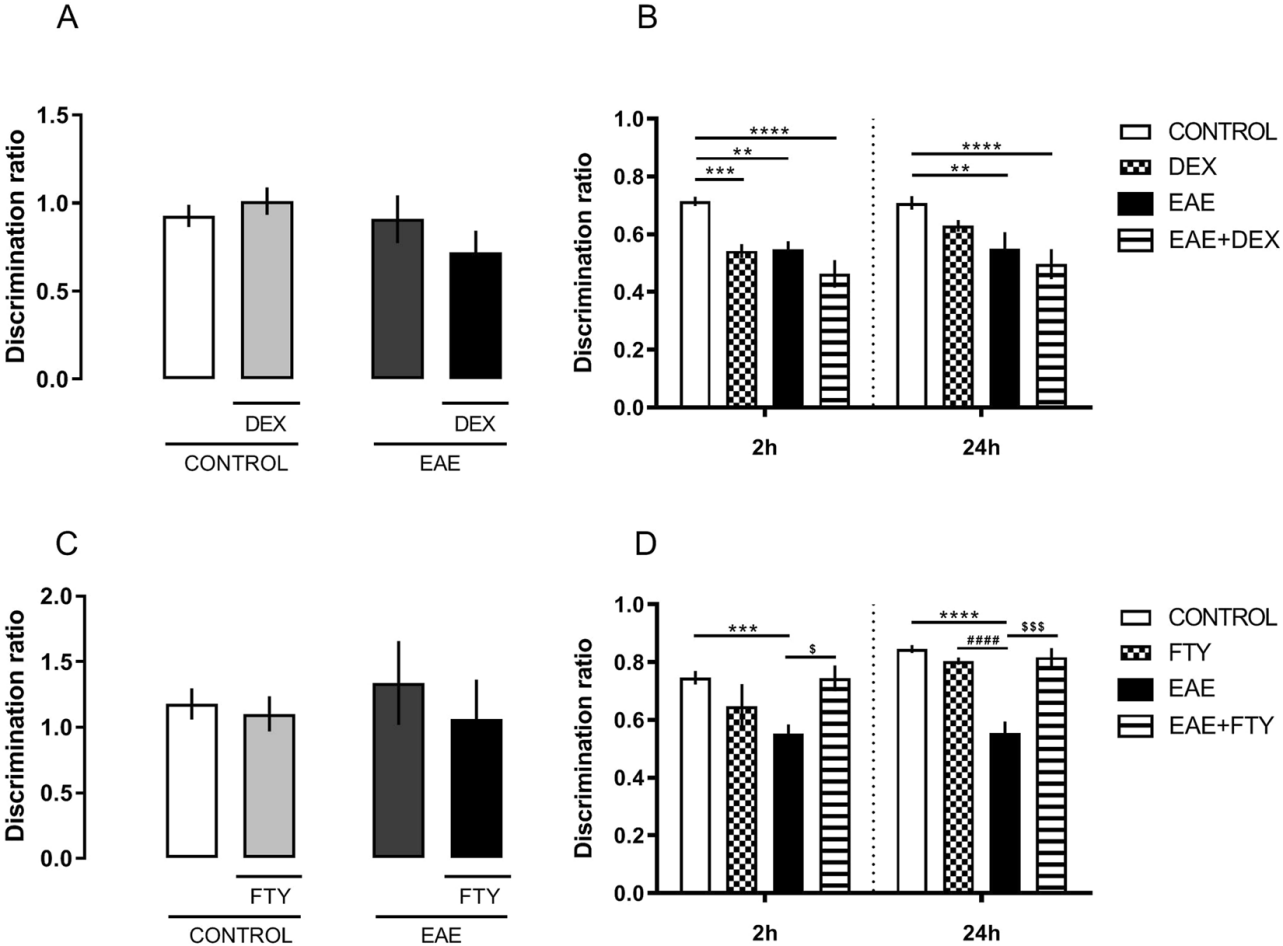
Fingolimod but not dexamethasone protects against memory loss after EAE induction at the 8 dpi, on Recognition Novel Object Task. (**A**) No differences in discrimination ratio were found among groups at the 8 dpi, the training day. (**B**) EAE and EAE + DEX groups showed decreased memory consolidation 2 and 24 hours after training. (**C**) No differences were founded in discrimination ratio in all groups after Fingolimod (FTY-720) treatment, 8 dpi. (**D**) Treatment with FTY-720 protects against working memory impairment promoted by EAE 2 and 24 hour after training at 8 dpi. Results are shown as mean + SEM of the experimental groups. *p < 0.05 vs control; ^#^p < 0.05 vs DEX or FTY-720. Two-way ANOVA test followed by the post-test Bonferroni, n = 6 to 12 for each group.

Using the Novel Object Recognition test (NOR), we addressed DEX modulation of learning and memory in EAE mice. In our experimental conditions, at the 8 dpi, both EAE and EAE + DEX groups showed decreased working memory consolidation in the EAE group, measured 2 and 24 hours after training, (DEX factor, *F (3,55)* = *21,51; P* < *0,0001*, Fig. 3A,B).

**Figure 4:**
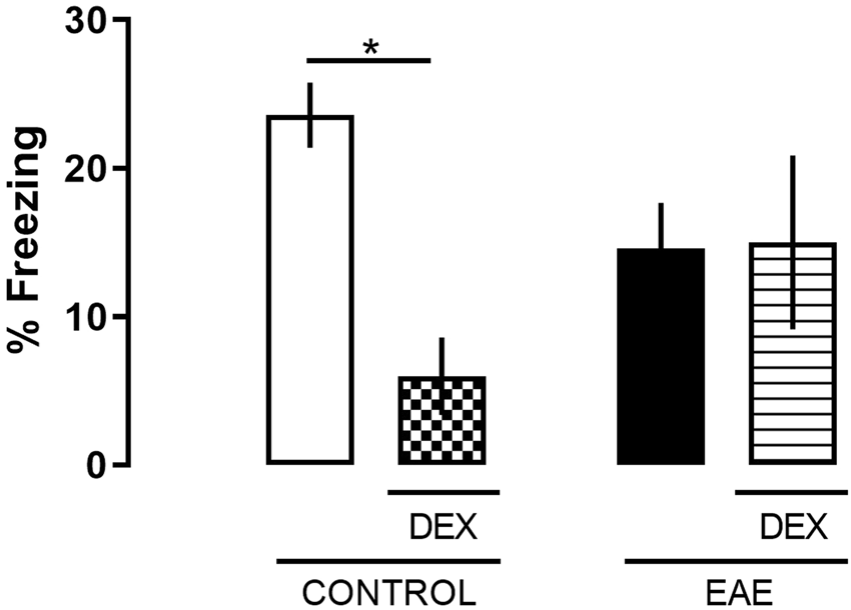
EAE did not change aversive memory acquisition in fear conditioning to context. Mice were trained at 7 dpi in a particular context. Next day (8 dpi), mice were placed in the same context, and their freezing responses were measured for the 5 minutes. Differences in freezing were found among control and DEX groups at the 8 dpi. The results are expressed as the mean ± SEM of the mean of the experimental groups. *p < 0.05 vs control. Two-way ANOVA test followed by the post-test Bonferroni, n = 5 and 7 for each group.

To confirm that DEX specifically caused the cognitive impairment, another set of animals receveid fingolimod (FTY-720), a sphingosin1-phosphate receptor modulator, 10 mg/kg, the first FDA-approved oral treatment for MS, on the day of immunization. In contrast to DEX treatment, fingolimod protected against memory loss in NOR in the same settings that DEX worsened it, 2 and 24 hours after training (interaction, F = (*1,13)* = *29,66;* P = 0,0001), and also reduced the clinical scores in EAE-treated mice (data not showed) (Fig. 3C,D). In our settings, EAE did not impair the contextual fear memory, suggesting that the acquisition of aversive memories is not affected after 8 dpi (Fig. 4).

### DEX decreased neuronal activity via GR signaling in the dorsal hippocampus of EAE mice in the absence of motor signs (8 dpi)

**Figure 5:**
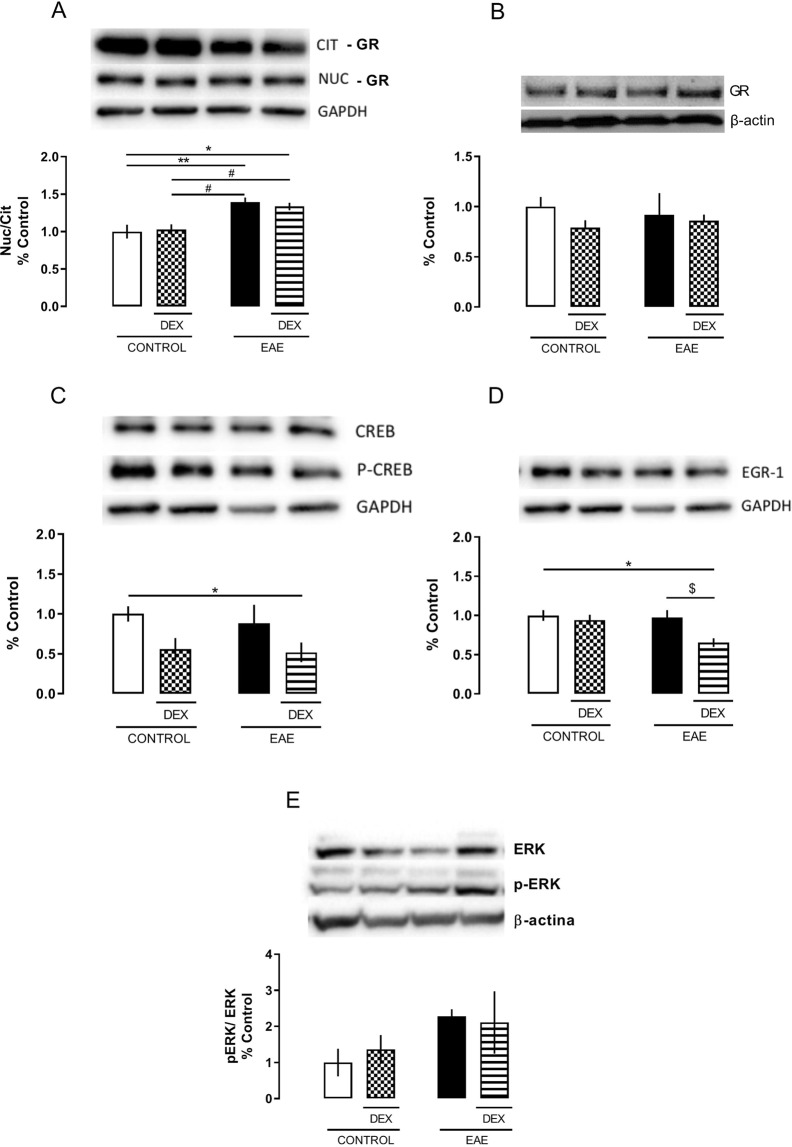
Dexamethasone treatment increases cytoplasmatic GR and decreases EGR-1 expression, at the 8 dpi, in the EAE dorsal hippocampus of C57BL/6 female mice immunized with MOG35-55 plus CFA, treated (DEX + EAE) or not (EAE) with dexamethasone (DEX, 50 mg/Kg) on the day of immunization. Western Blot analysis of proteins in the cytosolic and nuclear fractions. Upper panel: Representative image of the Western blot assay. Bottom panel: densitometric analyzes of the specific bands represented in the upper panel. (**A**) Glucocorticoid receptor (GR) translocation expressed by the nuclear and cytosolic GR expression ratio. (**B**) Total expression of GR. (**C**) CREB activation expressed by the ration of the phosphorylated (pCREB) and total (CREB) forms of the transcription factor CREB in nuclear extracts. (**D**) Nuclear EGR-1 protein expression in nuclear extract. (**E**) ERK activation expressed by the ration of phosphorylated (pERK) and total (ERK) ERK cytosolic expression. The results are expressed as the mean ± SEM of the mean of the experimental groups. *p < 0.05 vs control; ^#^p < 0.05 vs DEX; and ^$^p < 0.05 vs EAE. Two-way ANOVA followed by Bonferroni post-test, n = 4 for each group.

EAE cognitive effects can be associated with pro-inflammatory transcription factors activation in the hippocampus. Initially, we investigated the GR activity, using its nuclear translocation as an index of activation. EAE animals, even in the absence of clinical signs, showed increased GR translocation in the dorsal hippocampus when compared to control groups, an effect that is not modulated by DEX treatment (EAE *factor*: *F (1, 12)* = *25,17; P* = *0,0003*) (Fig. 5A). No differences were found in total GR expression (total protein extract) in the hippocampus among the groups (Fig. 5B).

Considering that the decrease in learning/memory acquisition could be related to disrupted hippocampal functions, we investigated the activity of the protein kinase ERK1/2, the transcription factor CREB, and the immediate early gene EGR-1, the latter as an indicator of neuronal activity. There was no difference in the ERK1/2 activity in the dorsal hippocampus among groups, measured by the ratio between the phosphorylated (Ser 133) and total ERK isoform expressions (Fig. 5E). However, DEX treatment, irrespective of the disease, decreased CREB phosphorylation in the serine 211 residue at the 8 dpi (*DEX factor (1, 11)* = *6,241* *P* = *0,0296*) (Fig. 5C).

**Figure 6:**
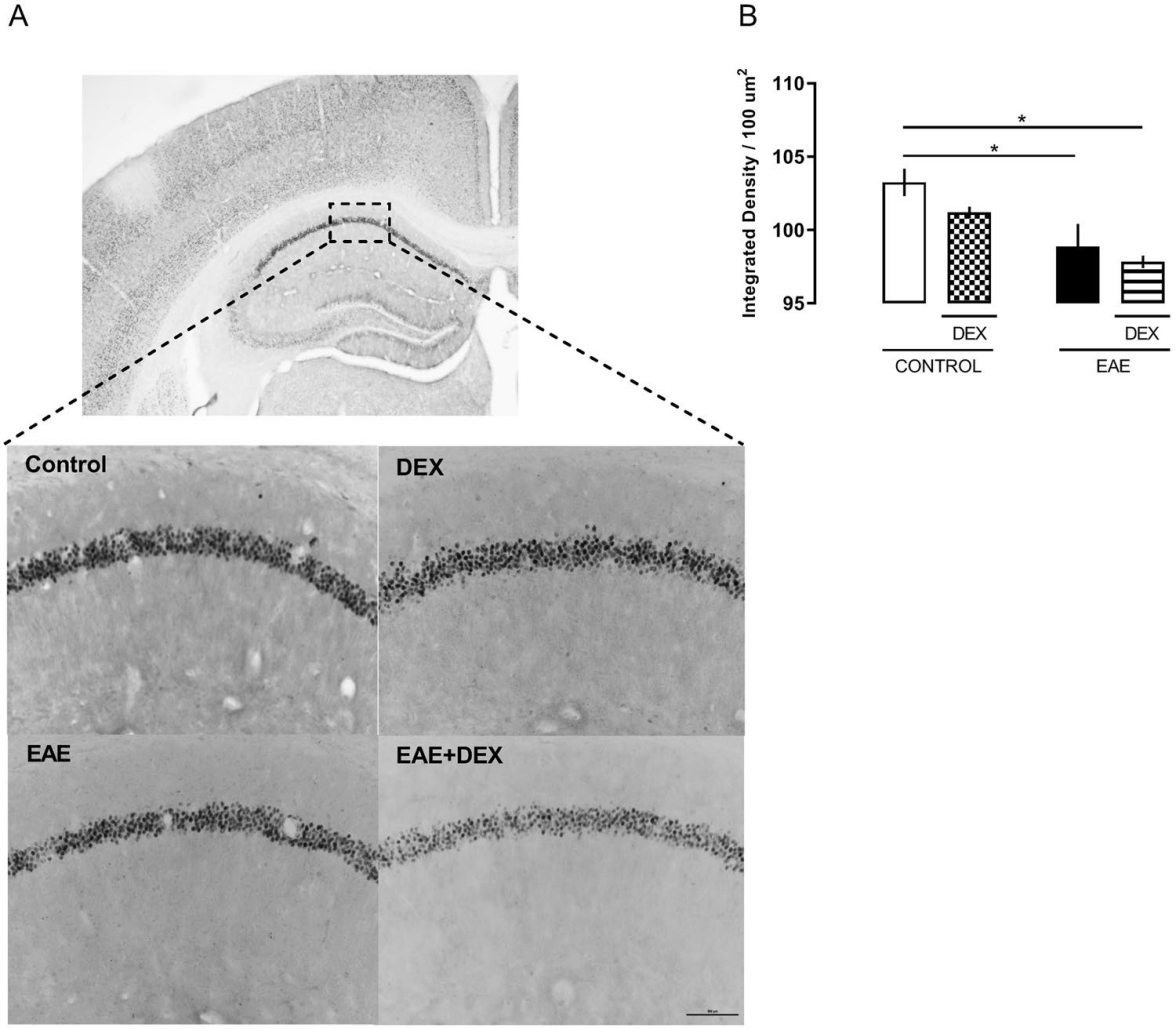
Dexamethasone did not protect against EGR-1 reduced expression, at the 8 dpi, in the EAE dorsal hippocampus of C57BL/6 female mice immunized with MOG_35-55_ plus CFA, treated (DEX + EAE) or not (EAE) with dexamethasone (DEX, 50 mg/Kg) on the day of immunization. (**A**) Representative photomicrographs (4x and 20x) of the immunohistochemistry assay. (**B**) Densitometric analyzes of the CA1 positive cells represented in the upper panel. Results are shown as mean ± SEM. *p < 0.05 vs control. Two-way ANOVA followed by Bonferroni post-test, n = 3 to 4 for each group.

CREB phosphorylation on Ser 211 residue can increase its binding to DNA and promotes the transcription of immediate early genes, such as EGR-1. Here, DEX treatment diminished the expression of EGR1, measured after NOR test, in both western blot analysis (*F (1, 12)* = *6,378; P* = *0,0266*) (Fig. 5D) and immunohistochemistry (*F (1, 9)* = *17,50; P* = *0,0024)* (Fig. 6A,B) of CA1 of dorsal hippocampus only in those animals with EAE, in agreement with the behavioral data obtained at the 8 dpi.

### Overexpression of dominant negative form of GR protected against learning/memory disruption in EAE animals at 8 dpi

**Figure 7:**
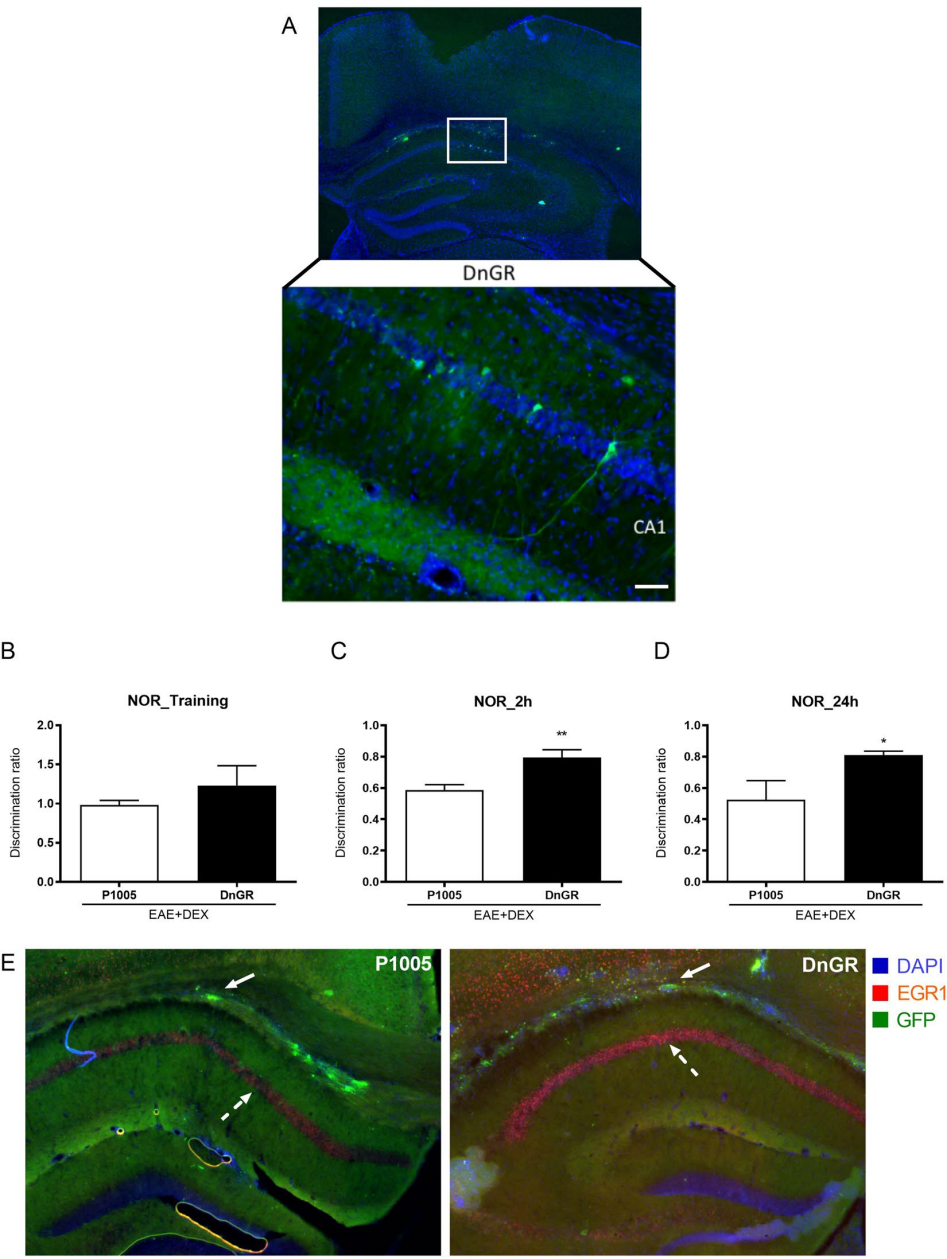
DnGR hippocampal CA1 administration reversed memory impairment in EAE + DEX animals at the 8 d.p.i. (**A**) GFP positive neurons in CA1 infected with DnGR. (**B**) Both p1005 and DnGR mice did not show differences in the discrimination ratio in the training day, at the 7 d.p.i. (**C**) Hippocampal DnGR overexpression protected against the EAE- induced decrease in memory consolidation 2 and (**D**) 24 hour after training in NOR test. EGR1 expression in dorsal hippocampus of EAE + DEX transfected with DnGR. Full arrows represent HSV-GFP transfection in fibers of hippocampus. Dashed arrows represent EGR-1 positive cell in CA1 of hippocampus. Representative photomicrographs (4x and 20x) of the immunofluorescence assay. Results are shown as mean + SEM. *p < 0.05 vs EAE + DEX/p1005 group. Mann-Whitney non-parametric test, n = 6 for each group.

To verify the role of GR in DEX-induced cognitive deficits in EAE mice, we overexpressed the DnGR transgene in the hippocampus of EAE-induced mice. The overexpression of the DnGR prevented the effects of DEX-induced cognitive impairment in EAE animals, confirming that GR can impair the learning and consolidation of working memory at 2 (*P* = *0,0087*) and 24 hours (*P* = *0,0152*) after training (Fig. 7B–D). In addition, and corroborating our hypotheses, EAE + DEX-P1005 group showed reduced EGR1 expression in the hippocampus compared to EAE + DEX-DnGR animals (Fig. 7E).

## Discussion

Neuroinflammation is critically involved in several disorders of CNS and understanding its mechanisms may evidence essential targets for the development of new therapies. MS and EAE usually present numerous pro and anti-inflammatory markers, which modulate the disease evolution^26,27^. Glucocorticoids are well known immunosuppressive drugs world-widely used in allergic diseases, organ transplantation, and autoimmune diseases such as MS^28^. The effectiveness of GC treatment in alleviating EAE-induced motor scores and peripheral inflammation is also showed in several settings^5,24^. In our paradigm, animals with EAE treated with dexamethasone (50 mg/kg) showed significant improvement of the clinical motor signs throughout the disease and decreased immune cells infiltrate in the spinal cord, evidencing the systemic immunosuppressive effect of GCs.

Although dexamethasone treatment has been protective against EAE motor signs progression in our study as in others, the role of GCs in neuroinflammation and cognition during EAE is quite controversial. Wurst and colleagues (2012) showed that methylprednisolone modulates the clinical signs after EAE immunization in a dose-dependent manner, with lower doses (20 mg/kg) being protective and higher doses (100 mg/kg, closest to the ones used in MS attacks) potentiating the motor deficits in ill animals^5^. These findings suggest that higher doses of methylprednisolone do not improve the treatment efficacy and may induce GC resistance by an HPA-axis dysregulation^5^ and the facilitation of alternative splicing of GR, generating two highly homologous isoforms: GRα and GRβ, this last one transcriptionally ineffective in humans^29^.

Unlike endogenous GCs, synthetic ones, such as dexamethasone, cannot be inactivated by the corticosteroid 11-beta-dehydrogenase isozyme 2 (11β-HSD2) and do not bind the corticosteroid-binding globulins, resulting, therefore, in an increased half-life. In addition, synthetic GCs such as dexamethasone crosses the blood-brain barrier, acting directly on neural cells^30^. In this context, synthetic GCs can exert their effects and promote HPA axis dysregulation with adverse effects such as the accumulation of fat in the extremities, rashes, hypertension, muscle atrophy, and neurological effects such as depression, anxiety and memory impairment^31^. A recent study from our group revealed that rats exposed to two-hour restraint stress exhibited anxiety-like behavior 10 days after stress, with no difference in serum corticosterone levels, evidencing that glucocorticoids induce long-lasting plasticity in the brain and modifies the behavior^32^. Also, Mitra and Sapolsky 2008, showed that a single administration of corticosterone increased anxiety and dendritic hypertrophy in the basolateral amygdala of rats 12 days after CORT administration but not before^33^. Moreover, stress and glucocorticoids changes spine and synapse density in the prefrontal cortex and hippocampus, as well. In the hippocampus, acute stress deeply affects glutamatergic transmission^34^.

GCs’ pro-inflammatory effects in the brain are well established and are dose-dependent. Higher concentrations administration, similar to the doses used to treat chronic inflammatory diseases (e.g., arthritis), stroke-induced edema or immune-related diseases (e.g., MS) increases, in several experimental settings, the levels of pro-inflammatory cytokines, as well as the NFKB activation in the hippocampus and frontal cortex of rats (for review see^35^). Furthermore, GR from both neurons and glia may contribute to pro- or anti-inflammatory effects of GC in subventricular brain regions. Hippocampal CA1 area, for example, presents high levels of GR^30^ and excitatory neurotransmitters (e.g., glutamate), being a brain structure highly susceptible to GC’s effects and excitotoxicity, respectively.

In our EAE paradigm, DEX modulated the HPA axis throughout the disease. First, corticosterone release was suppressed for at least 48 hours after DEX administration and returned to control levels after 8 days. Also, DEX treatment delayed CORT serum peak in EAE-induced mice. This late HPA activation differentially modulated GR and MR functions in the hippocampus, one of the brain regions implicated in memory processes^30,36^. In our study, DEX increased GR activity before the appearance of clinical signs, without changing MR signaling (data not shown).

Cognitive impairment and mood disorders are present in MS and EAE^14,37^ and glucocorticoids are well known to modulate cognition^38,39^. Nonetheless, this is the first study showing that DEX treatment, the gold standard for MS attacks control, did not protect against memory loss in EAE-induced mice and induced working memory deficits in EAE mice. Also, DEX treatment decreased acquisition in both working and aversive memories, evidencing the role of HPA axis in cognition. The effects of GCs on memory is also controversial, and several studies showed that the dose-dependent administration of glucocorticoids increased memory consolidation after training. However, high doses of GC without noradrenergic activation can impair the memory acquisition and consolidation^40^.

This effect was DEX-specific since fingolimod treatment (FTY-720), in the same settings that DEX, improved memory consolidation in EAE-animals in the asymptomatic phase. Fingolimod is a modulator of sphingosine 1-phosphate receptor, capable to promotes this receptor internalization and degradation^41^. Also, it is the first FDA-approved oral treatment for MS. In the peripheral system, this immunomodulator inhibits cell proliferation and infiltration in the CNS by decreasing STAT3 signaling and consequent inhibition of TH17 lymphocytes^42^. Several studies showed that FTY-720 promotes BDNF release^43^ and the elevation of neural stem cells in the hippocampus of mice via MEK/ERK signaling^44^. In contrast, early-life DEX and CORT exposition reduces CREB-BNDF-TrkB signaling in the hippocampus and promotes cognitive impairment in rats^45,46^. Differences between GCs and FTY-720 in non-aversive memory consolidation can be associated with the activation of different intracellular pathways in neurons^47^ and glial cells^19,20^.

In EAE models, neuronal death can be increased by retrograde neurodegeneration, decreased neurogenesis, and neuroinflammation. Di Fillippo and colleagues (2016) showed that IL1-β could disrupt EPSC amplitude in EAE hippocampal slices mediated by microglia and Acharjje *et al*., (2013) found that EAE impairs different types of memory in animals before the onset of clinical signs via microglial activation and pro-inflammatory cytokine mRNA in the hypothalamus. Altogether, these data corroborate our findings, where EAE-induced animals treated with dexamethasone showed working memory impairment and reduced neuronal activity measured by EGR-1 expression and CREB activity in the hippocampus.

CREB can be activated by phosphorylation via the MAP kinase pathway, resulting in its nuclear translocation and transcription activity of proliferative and immediate early genes such as *bdnf*, *egr-1* and *c-fos*^48^. Several studies showed correlations between EGR-1 expression and LTP maintenance in the hippocampus, with repercussion in learning of different tasks^49,50^. Furthermore, male mice exposed to chronic unpredictable stress showed reduced levels of *Egr1* mRNA in the hippocampus associated with cognitive impairments in the Morris water maze, novel object recognition and location tasks associated to CA1 dendrites atrophy^51^.

In this study, we found that DEX decreased the memory acquisition in non-aversive test (NOR), a phenomenon dependent on hippocampal activity. Whereas, there is no significant decrease in the acquisition of aversive contextual memory in the EAE groups. In accord, Acharjee and collegues 2013 suggests that release of cytokines in EAE impairs memory extinction but not acquisition^20^. In our study, EAE + DEX group also presented reduced levels of EGR-1 protein expression in the dorsal hippocampus, suggesting that the GR genomic action can decrease neuronal activity in the CA1 after 8 dpi, in an inflammatory environment. Also, overexpression of the dominant negative form of GR in the hippocampus reversed the learning and memory deficits in DEX-treated EAE animals at the asymptomatic phase, confirming that GR transcriptional activity in an inflammatory environment is essential for the cognitive impairment in EAE. It is, therefore, imperative to know whether this modulation depends on the other areas of the limbic system and glial cells, which have a crucial role in neuroinflammation and synaptic plasticity^52,53^.

In summary, dexamethasone showed distinct actions in periphery and CNS, decreasing immune activation and protecting against clinical sings progression of EAE, as well as increased inflammation in CNS via GR signaling, inducing memory/learning impairment and decreasing hippocampal neuronal activity in EAE mice. Altogether, our data suggest that glucocorticoid treatment, although has beneficial effects on the suppression of the immune system, thus, reducing the acute peripheral immune activation and further CNS infiltration, it seems to be crucial to the induction of cognitive impairment in some auto-immune, inflammatory conditions. Moreover, strengthening the current literature about the pro-inflammatory GC effects, in our EAE paradigm, GR signaling contributed not only to the maintenance of inflammation but also decreased neuronal activity caused by EAE in the mice CA1 hippocampi. Further studies are needed to elucidate whether and which post-translational and non-canonical mechanisms are involved in the pro-inflammatory GCs actions. Also, it is necessary to investigate the involvement of glial cells in this context, since these cells express GR, are widely known as modulators of neuronal activity, and have a crucial role in the inflammation of the CNS.

## Materials and Methods

### Chemicals and kits

Bradford Protein Assay Kit was obtained from BioRad (Hercules, CA, USA). Corticosterone EIA Kit was obtained from Enzo Life Sciences Inc (Farmingdale, NY, USA). MOG_35-55_ was obtained from Proteimax Technology (São Paulo, SP, BR). *Mycobacterium tuberculosis* and dexamethasone 21-phosphate disodium salt were obtained from Sigma Aldrich, St. Louis, MO, USA. Unless otherwise stated, the chemicals were from Sigma Aldrich.

### EAE induction

All experimental procedures, including immunization, infusion, and decapitation of animals, were approved and performed according to the standards of the Ethics Committee for Animal Use of the Institute of Biomedical Sciences/University of São Paulo (CEUA/ICB-USP). The procedures are by the guidelines of the Brazilian National Council for the Control of Animal Experimentation (CONCEA), under the Brazilian National Law number 11794 from 10/08/2008, which regulates all research activities involving animal use in the country. All efforts were made to minimize the number of animals used and their suffering.

EAE was induced as previously described by Inoue *et al*.^54^, with some modifications. On the day of immunization (day 0), C57BL/6 female mice were subcutaneously injected with 150 μg of MOG_35-55_ peptide emulsified in CFA (v/v) with 400 μg of heat-killed *Mycobacterium tuberculosis*. Also, 200 ng of *Bordetella pertussis* toxin was administered intraperitoneally (I.P.) twice, at 0- and 48-hours post-immunization. The acute high dose of Dexamethasone - DEX (50 mg/kg)^23^ or Fingolimod - FTY (10 mg/kg) was administrated (I.P.) on the immunization day. The animals were evaluated daily based on clinical scores (described below) to address the clinical signs of EAE and underwent euthanasia either by decapitation or transcardially perfusion under isoflurane anesthesia on the 8-day post immunization (dpi), corresponding to the pre-clinical phase of the disease. Control, no-EAE animals were inoculated with PBS emulsified in CFA.

Animals were evaluated daily, and the degree of clinical signs was distributed as follows: (0) without disease; (0.5) loss of tail tonus; (1) hind limb weakness; (2) One hind limb paralyzed; (3) Complete hind limbs paralysis; (4) Hind limbs paralyzed, weakness in forelimbs and (5) tetraplegia or death.

### Novel Object Recognition Test (NOR)

The NOR test analyzes the capacity of the animal to recognize a novel object based on the rodent’s natural tendency to explore new objects in their environment. Here, we used a protocol based on Leger at al, 2013 with few modifications^55^. Initially, in the habituation phase, each animal could explore the open field arena for 3 consecutive days from the 4 dpi to the 6 dpi. On the 7 dpi, in the training phase, each mouse was placed in the open field arena, which contained two identical objects (A and B), and allowed free exploration, for 10 minutes. Two (7 dpi) and 24 hours (8 dpi) after training, the object B was replaced by another (C and D, for short-term memory [STM] and for long-term memory [LTM], respectively) and the animals were returned to the open field arena for another 5 minutes to test the STM and LTM. Exploration was considered when the animals sniffed or touched the object with their nose or forepaws. A dim light illuminated the arena homogeneously. All trials were videotaped and, to avoid olfactory clues, the maze was cleaned with 5% (vol/vol) ethanol after each trial. Discrimination ratio in the training session was expressed by the ratio of the time exploring the right object and time exploring the left object (TR/TL). Discrimination index after the test sessions was expressed by the ratio (TN)/(TN + TF) [TN = time exploring the novel object, TF = time exploring the familiar object]. The experiments were conducted at the 7 dpi, characterized by the high peripheral inflammation without motor symptoms.

### Contextual fear conditioning

A fear conditioning experiment was carried out for two days in 22 cm × 22 cm × 20 cm boxes. On day 1, a mouse was placed in a conditioning chamber for 2 min, where, after this, it received an electric shock (0,5 mA, 1 s). On day 2, the mouse returned to the same box for 10 min. Freezing was defined as the lack of all movement of the body and vibrissae. A video camera recorded each experiment and the freezing time was analyzed during the first 5 min. The box was cleaned with 5% ethanol between each trial. Each video was renamed and analyzed blinded by two different evaluators.

### Serum corticosterone concentration

Serum corticosterone (CORT) was measured by Corticosterone EIA kit (Enzo Life Sciences, Inc), according to the manufacturer’s instructions. Serum was obtained from trunk blood. After collection, trunk blood samples could clot at room temperature for 30 min. The blood was then centrifuged at 4,000 rpm for 10 min, and the serum was transferred to a clear tube and stored at −80 °C until CORT analysis was conducted. Serum samples were diluted 1:30 and processed following manufacturer’s instructions.

### Immunohistochemistry for EGR-1

EGR-1 immunohistochemistry was used to assess neuronal activation in the CA1 and the dentate gyrus (DG) of the hippocampus of control, DEX, EAE, and DEX-treated EAE groups. Animals were transcardially perfused with ice-cold saline 0.9%, followed by ice-cold 4% paraformaldehyde in 0.1 M phosphate buffer pH 7.4. The fixed brains were removed from the skull and cryoprotected in a solution of 30% sucrose in 0.1 M phosphate buffer pH 7.4 at 4 °C. They were then frozen on dry ice and sectioned in the coronal plane at 40 μm thickness in a Leica semi-automatic cryostat (model 1850 UV, Leica Microsystems, Wetzlar, Germany).

Free-floating sections were blocked with blocking serum (3% donkey serum + 0,3% Triton X-100 in PBS) for 2 hours at room temperature. Subsequently, the sections were incubated with primary antibody Anti-EGR-1, 1:2000 (Santa Cruz Biotechnology, Dallas, TX, USA) diluted in blocking serum, overnight at 4 °C. After incubation with the primary antibody, the sections were incubated with the secondary antibody (goat anti-rabbit IgG, Vector, Burlingame, CA; dilution 1:2000) diluted in PBS + 0,3% Triton X-100 for 2 hours at room temperature, protected from light. The antigen-antibody complex was visualized using the avidin-biotin complex (Elite kit; Vector: dilution 1:200), and 3,3′-diaminobenzidine (DAB) was used as a chromogen. The peroxidase reaction product was developed using the glucose oxidase procedure^56^. The brain sections were mounted on gelatin-coated microscope slides, air-dried, dehydrated through an ascending series of alcohols, cleared with xylene, and coverslipped with DPX mounting medium.

Positive cells were observed with a Nikon Eclipse 80i microscope (Nikon Instruments Inc., NY, USA). The images were captured through the Nikon Digital Camera DXM 1200 C and analyzed by NIS-Elements Advanced Research 2.30 Image Software (Nikon Instruments Inc.). Eight boxes of 100 µm^2^ were used to measure the integrity density in the granular layer of CA1 of the dorsal hippocampus. The mean of four sections of each animal was used to determine differences between experimental groups.

### Tissue samples preparation

Nuclear and cytosolic protein extracts were prepared using the CelLytic™ NuCLEAR™ Extraction Kit (Sigma-Aldrich Co.), as briefly described below. Dorsal hippocampi were homogenized in cold lysis buffer (10 mm HEPES, pH 7.9, 1.5 mm MgCl2, 10 mm KCl, 1 mm DTT, 0.5 mm PMSF) using a Dounce homogenizer and centrifuged at 4 °C for 20 min at 11,000 × g. The supernatants, used as the cytosolic fraction in the Western blot assays, were stored at −80 °C. The pellets, containing the nuclear fraction, were resuspended in extraction buffer (20 mm HEPES, pH 7.9, 25% glycerol, 1.5 mm MgCl_2_, 300 mm NaCl, 0.25 mm EDTA, 0.5 mm DTT, 0.5 mm PMSF), kept on ice for 30 min, and centrifuged at 4 °C for 5 min at 20,000 × g. All buffers were complemented with the Halt™ Protease and Phosphatase Inhibitor Cocktail (Thermo Fisher Scientific, Inc.). The resulting supernatants containing nuclear proteins were stored at −80 °C. Protein concentration was determined using the Bradford method (BioRad Laboratories, Inc.)^57^. For the total extract, dorsal hipocamppi were sonicated in a cold homogenization buffer (Tris-HCl 20 mM - pH 7.5; EDTA 1 mM; DTT 1 mM; PMSF 1 mM; leupeptin 2 μg/μl; pepstatin 1 μM). The pellet was removed by low-speed centrifugation at 1,100 × g × 20 min at 4 °C. The resulting supernatants containing nuclear proteins were stored at −80 °C. Protein concentration was determined using the Bradford method (BioRad Laboratories, Inc.)^58^.

### Western blot assay

Electrophoresis was performed using a 10% polyacrylamide gel and the Mini-Protean® Tetra Cell apparatus (BioRad Laboratories, Inc.). The proteins present in the cytosolic and nuclear fractions were combined with an equal or a quarter part of the supernatant volume with Laemmli’s buffer (BioRad Laboratories, Inc.; complemented with 5% 2-mercaptoethanol) and boiled at 95 °C for 5 min. Protein samples (20 µg/lane) were size-separated in 10% SDS-PAGE gel (90 V) and then blotted onto Immobilon® PVDF membrane (EMD Millipore Corporation). Ponceau method to immunoblot was used to ensure equal protein loading^59^. Blots were blocked with 5% non-fat milk or bovine serum albumin (BSA), for phosphorylated proteins, diluted in TBS-T buffer (50 mM Tris-HCl, 150 mM NaCl, 0.1% Tween 20, pH 7.5) for 1 hour at room temperature, and subsequently incubated overnight at 4 °C with specific antibodies: GR, EGR-1 (1:1000, Santa Cruz Biotechnology); ^Thr202/tyr204^phospho-ERK1/2, ERK1/2 (1:5000 Cell Signaling Technology); CREB and ^Ser133^phospho-CREB (1:1000, Cell Signaling Technology). After incubation with the primary antibodies, the membranes were then probed with a secondary antibody conjugated to horseradish peroxidase (dilution of 1:2000–1:4000, Kirkegaard & Perry Laboratories) for 2 hours at room temperature and developed by ECL-Immobilon® reagent (EMD Millipore Corporation). Chemiluminescent bands were quantitatively analyzed by optical density analysis using ChemiDoc MP Imaging System Detection System and Image Lab software (BioRad). Several exposure times were analyzed to ensure the linearity of the band intensities. The relative density of each band was normalized to the value of α-Tubulin, GAPDH or β-actin (dilution of 1:10000, Santa Cruz Biotechnology). The ratio between phospho-ERK-1/2 and total ERK-1/2 and CREB and phospho-CREB expression was used as phosphorylation index, and the ratio between the nuclear and cytosolic expression of GR was computed as the nuclear translocation index, and therefore, transcriptional activity.

### Hippocampal DnGR Viral injection

#### Amplicon construction of DnGR

DnGR was generated as previously described (Kaufer *et al*., 2004) and was kindly donated by Dr. Ki Ann Goosens (McGovern Institute for Brain Research, MIT, Cambridge, MA-USA). A bicistronic herpes simplex viral vector (p1005-HSV) was used to pack the HSV-GFP and DnGR vectors. Amplicons were generated using 5dl1.2 helper virus as described in^60^.

#### Mouse HSV Infusions and EAE induction

Mice were anesthetized with a ketamine/xylazine mixture (ketamine 100 mg/kg and xylazine 10 mg/kg) and prepared for stereotaxic surgery. Thirty-three-gauge syringe needles (Hamilton) were used to infuse 0.5 µl of virus bilaterally into the CA1 hippocampal region (1.8 mm anteroposterior and 1.5 mm lateral, from Bregma, and 1,2 mm dorsoventral from dura) at a rate of 0.1 µl/2 min. Animals could recover between 3 and 6 days (peak of viral expression) after HSV delivery and then were induced with EAE and treated with DEX as described earlier. Viral injection sites were confirmed by searching for the GFP signal in the brain slices using a fluorescence microscope.

### Experimental design and statistical analysis

Different behavioral tests were performed between 8 dpi as outlined in Fig. 1A. For clinical scores analysis, the data were treated as repeated measures, and the differences between the experimental and the control groups were detected by unpaired T-test. Two-way ANOVA was used when more than 2 experimental groups were analyzed followed by *post-hoc* Bonferroni test. In these situations, disease conditions (Control or EAE) and treatment (saline or DEX) were between-subject factors. The level of significance was set at a maximum of 5% (p < 0.05) for all tests. Data are presented as the mean ± standard error of the mean (SEM). The F values and experimental degrees of freedom (DF) are included in the Results session. All analyzes were performed using the GraphPad Prism7 software package, GraphPad Software, San Diego, CA, USA.

## Supplementary information

Western Blot Assays
