# Supplementary material for: High dose of dexamethasone protects against EAE-induced motor deficits but impairs learning/memory in C57BL/6 mice
Source: Sci Rep. 2019 Apr 30;9:6673. doi: 10.1038/s41598-019-43217-3 (PMC6491620; doi:10.1038/s41598-019-43217-3)
Supplement: Supplementary file 1 — Western Blot Assays [file 41598_2019_43217_MOESM1_ESM.pdf]

High dose of dexamethasone protects against EAE-induced motor deficits but impairs learning/memory in C57BL/6 mice

[1]Nilton Santos, [1] Leonardo Novaes, [1] Guilherme Dragunas, [1] Jennifer Rodrigues, [2] Wesley Brandão, [1] Rosana Camarini, [2] Jean Pierre Schatzmann Peron and [1*]Carolina Demarchi Munhoz

[1] Department of Pharmacology, Institute of Biomedical Science, University of São Paulo, São Paulo, Brazil, 05508-000.

[2] Department of Immunology, Institute of Biomedical Science, University of São Paulo, São Paulo, Brazil, 05508-000.

*Corresponding author: Carolina D. Munhoz (cdmunhoz@usp.br) at Department of Pharmacology, Av. Prof. Lineu Prestes, 1524, room 323. Institute of Biomedical Sciences, University of Sao Paulo, Sao Paulo - SP, 05508-000, Brazil.

Supplementary data

Western Blot

To measure the nuclear translocation of GR, used as an index of its activation and the protein expression of EGR-1, $^{Thr202/tyr204}$phospho-ERK1/2, ERK1/2, and CREB and ^{Ser133}phospho-CREB, four SDS-PAGE gels containing two experimental samples from dorsal hippocampus of Control, DEX, EAE, EAE+DEX groups were used and blotted against GR, EGR-1 (1:1000, Santa Cruz Biotechnology); $^{Thr202/tyr204}$phospho-ERK1/2, ERK1/2 (1:5000 Cell Signaling Technology); CREB and ^{Ser133}phospho-CREB (1:1000, Cell Signaling Technology). Image lab software (Biorad) was used to detect the specific bands, applying a signal accumulation mode. For each membrane, 6 images were captured in a total of 10 seconds for GR and β-actin and 6 images in a total of 120 seconds to EGR-1; $^{Thr202/tyr204}$phospho-ERK1/2; ERK1/2; CREB; and ^{Ser133}phospho-CREB antibodies. The last photographed image before saturation was quantified using the adjacent volume. The analysis of β-actin (42 Kd) was performed in the same manner than the other antibodies. Furthermore, only adjustments in brightness and contrast were perform in the images.

The figure S1 represents the two membranes from Western Blot assays showing the cytosolic GR protein expression in the dorsal hippocampus of control, DEX, EAE, and EAE+DEX groups (n=2 per membrane).

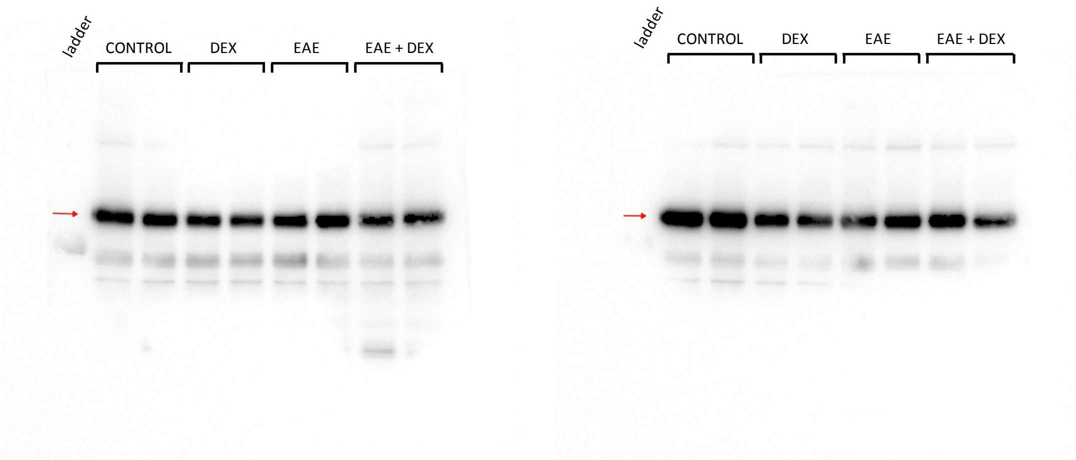

Figure S1. Western blot analysis of cytosolic GR protein expression in the dorsal hippocampus of control, DEX, EAE, EAE+DEX. The arrow indicates the 90 kDa band for GR.

The figure S2 represents the two membranes from Western Blot assays showing the nuclear GR protein expression in the dorsal hippocampus of control, DEX, EAE, and EAE+DEX groups (n=2 per membrane).

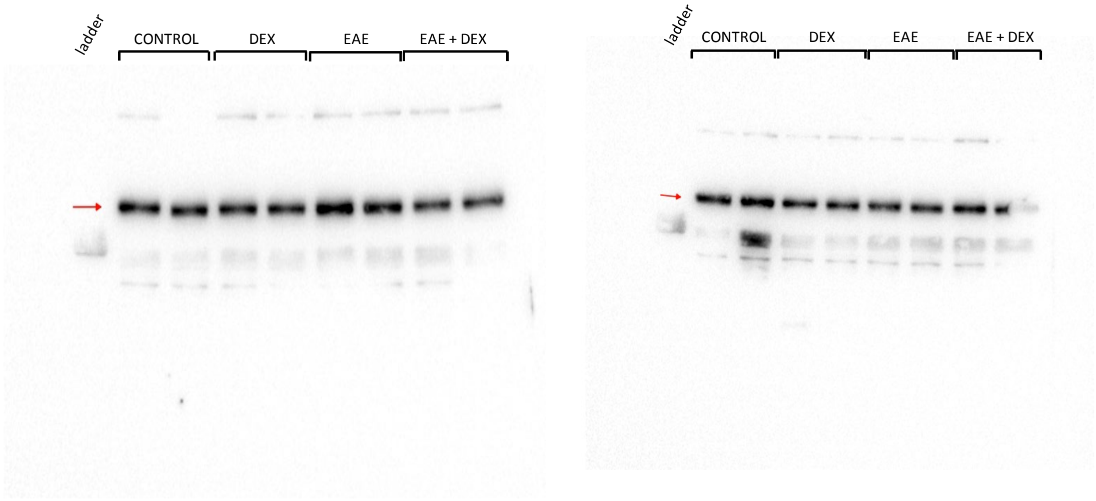

Figure S2. Western blot analysis of nuclear GR protein expression in the dorsal hippocampus of control, DEX, EAE, EAE+DEX. The arrow indicates the 90 kDa band for GR.

The figure S3 represents the two membranes from Western Blot assays showing the cytosolic β-actin protein expression in the dorsal hippocampus of control, DEX, EAE, and EAE+DEX groups (n=2 per membrane).

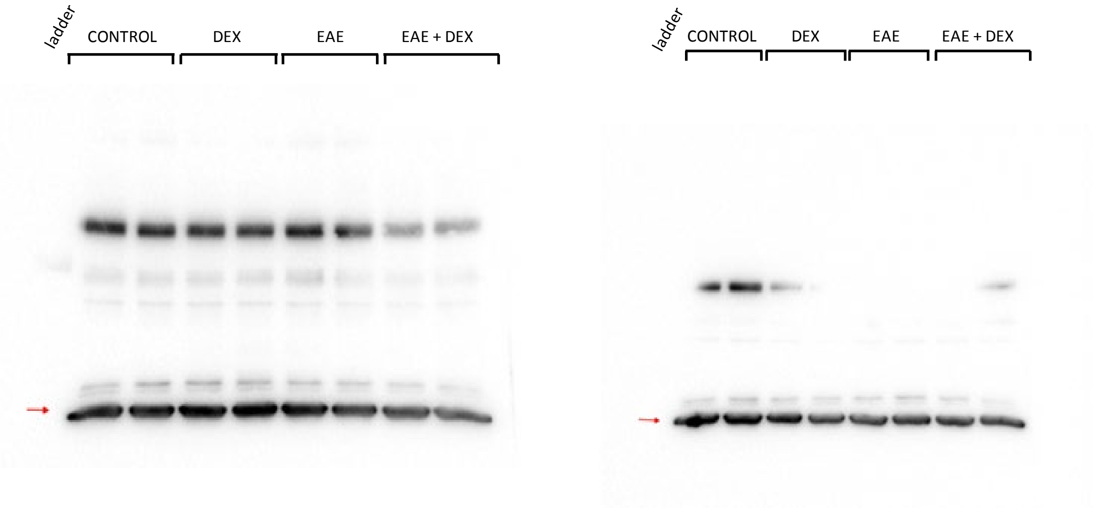

Figure S3. Western blot analysis of cytosolic β-actin protein expression in the dorsal hippocampus of control, DEX, EAE, EAE+DEX. The arrow indicates the 42 kDa band for β-actin.

The figure S4 represents the two membranes from Western Blot assays showing the nuclear β-actin protein expression in the dorsal hippocampus of control, DEX, EAE, and EAE+DEX groups (n=2 per membrane).

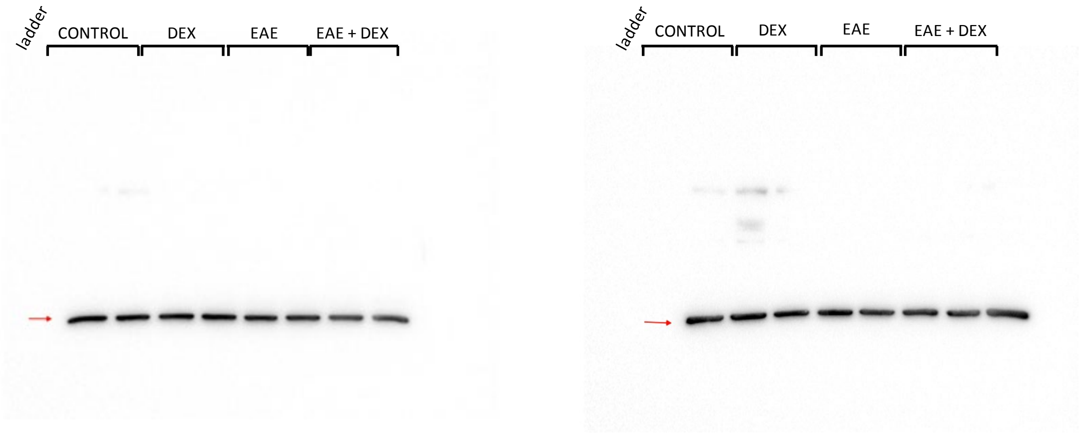

Figure S4. Western blot analysis of nuclear β-actin protein expression in the dorsal hippocampus of control, DEX, EAE, EAE+DEX. The arrow indicates the 42 kDa band for β-actin.

The figure S5 represents the two membranes from Western Blot assays showing the nuclear EGR-1 protein expression in the dorsal hippocampus of control, DEX, EAE, and EAE+DEX groups (n=2 per membrane).

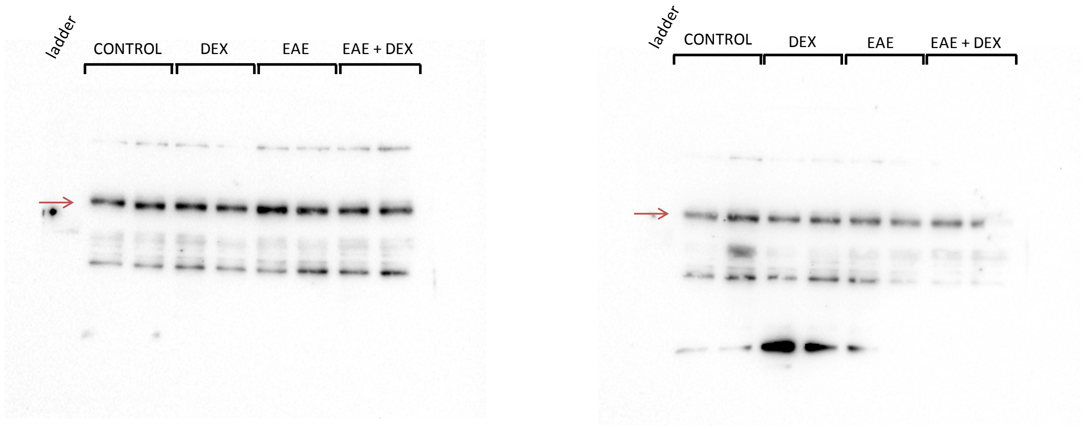

Figure S5. Western blot analysis of nuclear EGR-1 protein expression in the dorsal hippocampus of control, DEX, EAE, EAE+DEX. The arrow indicates the 80 kDa band for EGR-1.

The figure S6 represents the two membranes from Western Blot assays showing the nuclear CREB protein expression in the dorsal hippocampus of control, DEX, EAE, and EAE+DEX groups (n=2 per membrane).

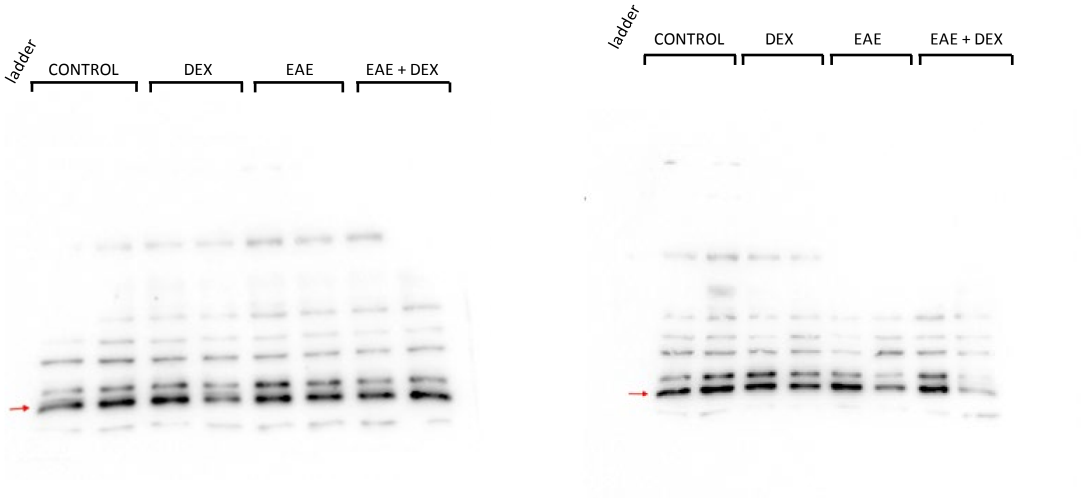

Figure S6. Western blot analysis of nuclear CREB protein expression in the dorsal hippocampus of control, DEX, EAE, EAE+DEX. The arrow indicates the 42 kDa band for CREB.

The figure S7 represents the two membranes from Western Blot assays showing the nuclear ^{Ser133}phospho-CREB protein expression in the dorsal hippocampus of control, DEX, EAE, and EAE+DEX groups (n=2 per membrane).

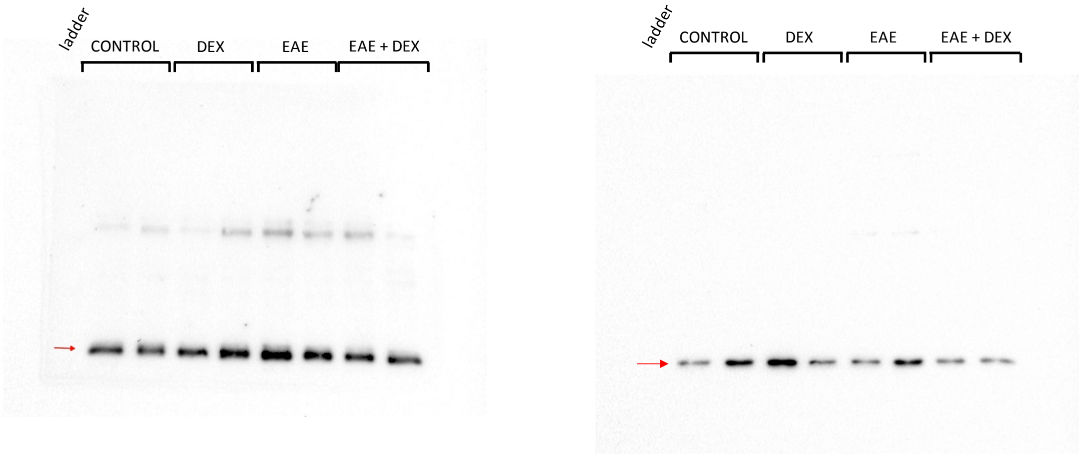

Figure S7. Western blot analysis of nuclear ^{Ser133}phospho-CREB protein expression in the dorsal hippocampus of control, DEX, EAE, EAE+DEX. The arrow indicates the 42 kDa band for ^{Ser133}phospho-CREB.

The figure S8 represents the two membranes from Western Blot assays showing the nuclear β-actin protein expression in the dorsal hippocampus of control, DEX, EAE, and EAE+DEX groups (n=2 per membrane).

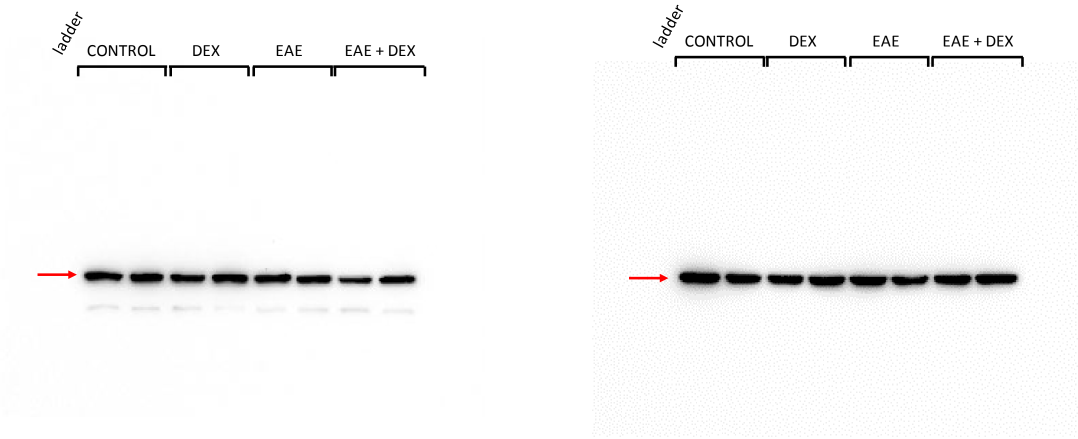

Figure S8. Western blot analysis of nuclear β-actin protein expression in the dorsal hippocampus of control, DEX, EAE, EAE+DEX. The arrow indicates the 42 kDa band for nuclear β-actin

The figure S9 represents the membranes from Western Blot assays showing the total ERK 1/2 (A) and β-actin (B) protein expressions in the dorsal hippocampus of control, DEX, EAE, and EAE+DEX groups (n=3 per membrane).

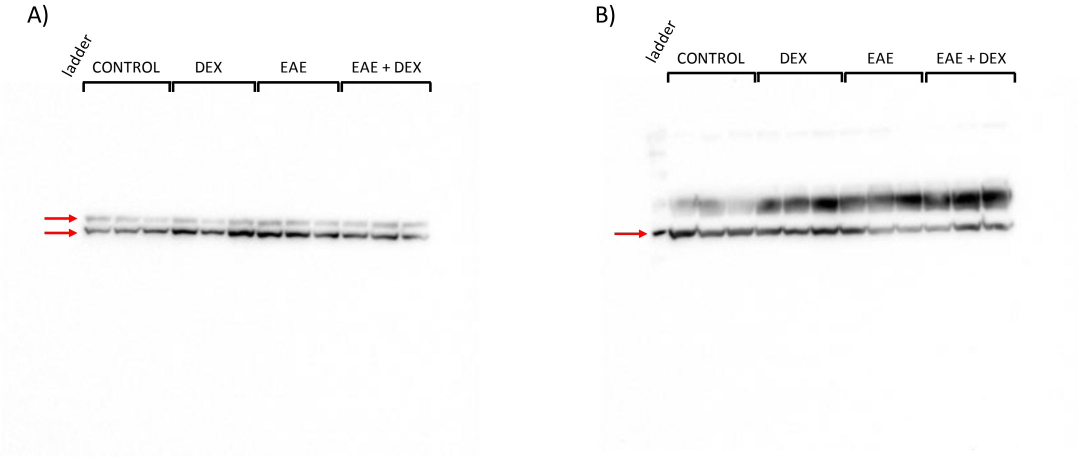

Figure S9. Western blot analysis of total ERK 1/2 (A) and β-actin (B) protein expression in the dorsal hippocampus of control, DEX, EAE, EAE+DEX. The arrow indicates the 44/42 kDa band for ERK 1/2 (A) and the 42 kDa band β-actin (B).

The figure S10 represents the membranes from Western Blot assays showing the $^{Thr202/tyr204}$phospho-ERK1/2 (A) and β-actin (B) protein expressions in the dorsal hippocampus of control, DEX, EAE, and EAE+DEX groups (n=3 per membrane).

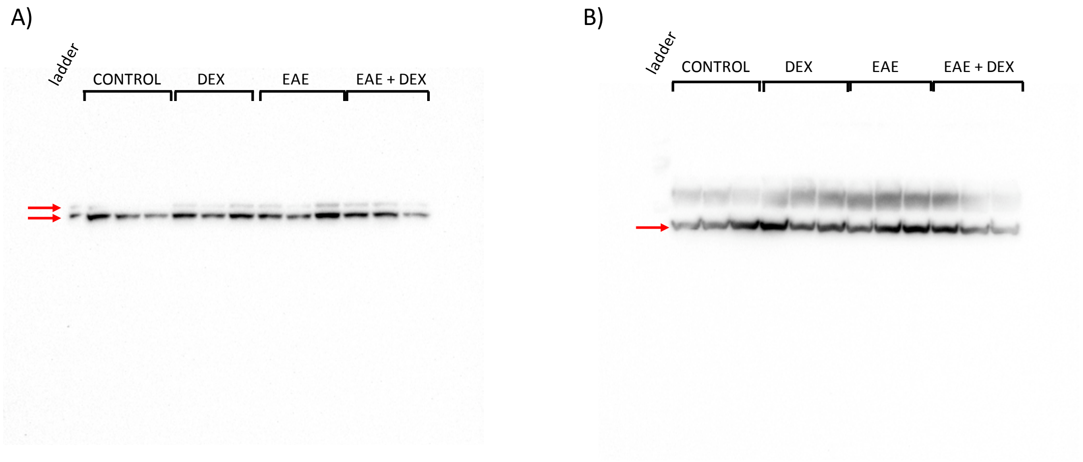

Figure S10. Western blot analysis of $^{\text{Thr202/tyr204}}$phospho-ERK1/2 (A) and β-actin (B) protein expression in the dorsal hippocampus of control, DEX, EAE, EAE+DEX. The arrow indicates the 44/42 kDa band for ERK 1/2 (A) and 42 kDa band for β-actin (B).
